# Genetic determinants of swallowing impairment, recovery and responsiveness to treatment

**DOI:** 10.1007/s40141-016-0133-6

**Published:** 2016-08-08

**Authors:** Alicja Raginis-Zborowska, Neil Pendleton, Shaheen Hamdy

**Affiliations:** 1 Centre for Gastrointestinal Sciences, Institute of Inflammation and Repair Faculty of Medical and Human Sciences, The University of Manchester, Manchester, UK; 2 Institute of Brain, Behaviour and Mental Health, The University of Manchester, Manchester, UK

**Keywords:** Dysphagia, Swallowing, Genes, Polymorphism

## Abstract

**Purpose of review:**

Here we review the latest literature and evidence in the field of genetics and determinants of swallowing and its treatments—specifically, this is a very recent concept in the field of oropharyngeal dysphagia, with only now an emerging research interest in the relationship between our genetic makeup and the effect this has on swallowing function and dysfunction. As such our review will look at preclinical, clinical and hypothesis generating research covering all aspects of the genetics of swallowing, giving new importance to the genotype-phenotype influences pertaining to dysphagia and its recovery.

**Recent findings:**

There appear to be a number of candidate gene systems that interact with swallowing or its neurophysiology, which include brain-derived neurotrophic factor, apolipoprotein E and catechol-*O*-methyltransferase, that have been shown to impact on either swallowing function or the brain’s ability to respond to neurostimulation and induce plasticity. In addition, a number of genetic disorders, where dysphagia is a clinical phenomenon, have given us clues as to how multiple genes or the polygenetics of dysphagia might interact with our swallowing phenotype.

**Summary:**

There is currently limited research in the field of genetic factors that influence (human) swallowing and oropharyngeal dysphagia, but this is an emerging science and one which, in the future, may herald a new era in precision medicine and better targeting of therapies for dysphagia based on an individual’s genetic makeup.

## Introduction

Dysphagia is a common symptom among “healthy” elderly and frail individuals. Increased numbers of cases with dysphagia are being observed among residents of care homes (up to 52.5 %) and hospital units compared with those who live in their own homes (from 13 %) [1, 2]. Other at-risk groups include patients with neurodegenerative disorders such as Parkinson’s disease (with up to 85 % incidence of dysphagia) [3], patients with stroke (20–63 % incidence of dysphagia) [4, 5–7] and patients with chronic neurological conditions including multiple sclerosis, head injury, amyotrophic lateral sclerosis, and myasthenia gravis [8]. The discrepancies between prevalence data on dysphagia may result from a number of factors including patient demographic differences, method of identification and diagnosis of dysphagia and differences in study design such as interventional versus observational studies. Moreover, patients with dysphagia reveal different recovery patterns [7], which often impact on the effectiveness of existing therapies. The factors that influence both the propensity for dysphagia, its recovery and response to treatment remain unclear but it seems increasingly likely that an individual’s genetic make-up may play an important role in these processes. Indeed, one of the key drivers in recovery from swallowing impairments is change in neuronal excitability within the swallowing motor cortex [9]. Cortical neuronal excitability associated with swallowing performance remains an area of interest and again is thought to be influenced by genetic factors.

Hence, knowledge about the features of an individual’s genotype has the potential for developing novel treatment strategies within stratified medicine. Stratified medicine (also called personalised or precision medicine) is an approach which subdivides patients into groups based on their risk of developing specific diseases/symptoms or their response to particular treatment therapies.

There are a number of ways to explore complex disease/symptoms [10]. One of the first steps is classification of the disease/symptom as heritable, usually after conducting family or twin studies. Unfortunately, no twin studies on swallowing processes have been done to date. However, cortical excitability remains a significant avenue for exploration of genetic propensity. Twin studies have shown that cortical excitability (a plausible driver in the recovery of dysphagia following neurologic damage) induced by non-invasive brain stimulation delivered over the hand motor cortex might have a genetic component. The heritability estimate for brain motor excitability was 0.68, which means 68 % of the variance can be explained by genetics [11]. A similar phenomenon is likely to occur in the swallowing motor system.

The following review will therefore present the current state of knowledge on the genetic background of swallowing processes from both human and animal studies, where individual genes, single nucleotide polymorphisms (SNPs) or chromosomal regions have been examined. The existing literature highlights a number of single genes or SNPs which might take a part in the human and animal swallowing neurophysiology. Additionally, genetic syndromes, in which one of the features is dysphagia, will be explored. These studies provide information on chromosomal localisation of genes which may take a part in the process of impaired swallowing development. This review will mainly focus on oropharyngeal dysphagia rather than oesophageal impairments which most likely have different aetiologies.

## Genetics of swallowing in humans

One of the novel treatment approaches for swallowing problems is the application of (non-invasive) brain stimulation to influence the swallowing motor cortex and measure responses with motor evoked potentials (MEPs) and assess behaviour with videofluoroscopy (VFS) and/or questionnaires. One such technique is repetitive Transcranial Magnetic Stimulation (rTMS) where either high- or low-frequency regimens are used to modulate swallowing motor cortex. Studies on healthy volunteers showed that high-frequency rTMS delivered over the pharyngeal motor cortex increases excitability measured with MEPs [12, 13, 14••] and low frequency decreases the excitability [14••, 15]. The exact mechanisms of plasticity remain unknown; however, both frequencies were used in clinical trials to improve swallowing performance [9, 16–21].

Neurostimulation studies conducted on healthy volunteers provide a useful tool to explore the genetics of swallowing neurophysiology and reactiveness for treatments and recovery. Certain genes may predispose individuals to display expected or unexpected outcomes from stimulation. No studies have been done on stroke patients with regard to exploring their genetic predispositions to outcomes from rTMS interventions; however, studies conducted in healthy volunteers might provide information of potential genetic markers of swallowing neurophysiology.

The first study exploring the genetic basis of neurological control of swallowing was conducted by Jayasekeran et al. [14••]. The study focused on an SNP from the *BDNF* gene ((Online Mendelian Inheritance in Man (OMIM) number: 113505)). The main aim of the study was to find an association between Val66Met (rs6265) SNP and its impact on the pharyngeal muscle responses followed by 1 and 5 Hz rTMS paradigms and pharyngeal electrical stimulation (PES). The *BDNF* gene is located on the chromosome 11, locus 11p13 and is a member of the nerve growth factor family. *BDNF* is expressed by cortical neurons, and is necessary for survival of striatal neurons in the brain. Multiple studies showed that rs6265 from the *BDNF* gene affects cortical plasticity and motor responses in both healthy adults and patients with brain lesions [22–24, 25•, 26]. Polymorphism rs6265 located in the coding region of the *BDNF* causes substitution of valine (Val) to methionine (Met) in the codon 66.

Jayasekeran et al. [14••] showed a link between provoked neuronal plasticity of the pharyngeal area and the impact of the polymorphism rs6265. As an outcome, MEPs from the pharyngeal muscles were collected with an intra-luminal catheter placed in the individual’s throat. The individuals were divided into two groups according to their genotype from the codon 66 of *BDNF*: Val/Val and non-Val/Val (carrying Val/Met of Met/Met) groups. Statistical analysis showed significant differences between the pharyngeal MEPs in homozygous participants with Val/Val comparing to participants carrying at least one *BDNF* Met allele after 5 Hz rTMS. This study suggests the plausible hypothesis of a genetic factor in pharyngeal cortical plasticity. Jaysekeran’s et al. study was the first to use a human model of this nature to study swallowing neurophysiology and genetics. The main disadvantage of this research was the examination of the single-gene polymorphism, since the majority of common diseases are most likely multifactorial and polygenic (complex) that may include gene–gene or gene–environmental interactions [27].

Of relevance, *BDNF* rs6265 status may also affect oesophageal sensitivity induced by electrical stimulation. Another study by Vasant et al. [28] used electrical stimulation of the oesophagus of healthy subjects to measure sensitivity and its association with rs6265. The study explored the relationship between oesophageal sensitivity and *BDNF r*s6265 genotype and found that Met allele carriers were more likely to have lower levels of sensory tolerance to oesophageal electrical stimulation. The results were independent of self-reported anxiety and depression scores.

Preliminary results from Essa et al. [29] described a possible association between *BDNF* rs6265 and response to pharyngeal electrical stimulation (PES) in stroke patients’ population. The authors suggested that active PES in the presence of the *BDNF* Met allele might play a role in improvement of swallowing function at the 3-month stage as compared to non-Met allele.

Another field of research delivers a more comprehensive approach in exploring genetic basis of swallowing impairments. These are population studies where a more general phenotype of swallowing is used. Self-reported swallowing questionnaires about swallowing symptoms related to dysphagia are especially useful in the early screening of both patients and healthy individuals in the early stages of swallowing problems.

Mentz et al. [30] performed the first association analysis between self-reported swallowing symptoms from the (University of Manchester’s) Dyne Steel Cohort of heathy elderly volunteers and the *APOE* gene (OMIM number: 107741). The *APOE* gene encodes apolipoprotein which is essential for normal catabolism of triglyceride-rich lipoprotein constituents. It has been discovered that isoforms of *APOE* are related to neurological conditions and cognitive decline [31–33]. This study used a more global approach, assessing 634 elderly volunteers. Volunteers completed a self-reported Sydney Swallow Questionnaire (SSQ) concerning the presence of swallowing problems. The score was classified as clinically significant if the obtained value ≥120. The study showed that there was an association between *APOE* E4 homozygosity and higher scores from the SSQ questionnaire. The main advantage of the study was the number of individuals included, which gives a better statistical power of the result.

Another study conducted on 538 participants with self-reported swallowing problems was performed by Nimmons et al. [34]. The researchers again used the Dyne Steel cohort. However, the analysis explored two genes: 12 SNPs from *BDNF* and 18 SNPs from *COMT* gene. *COMT* gene (OMIM 116790; cytogenetic location: 22q11.21) encodes the protein which catalyses the inactivation of catecholamine neurotransmitters and catechol hormones. *COMT* protein shortens the biological half-lives of certain neuroactive drugs such as L-dopa [35]. Interestingly, the status of two interactive SNPS from *COMT* polymorphism rs165599 and the *BDNF* polymorphism rs10835211 was shown to predict dysphagia in this cohort of elderly individuals. This finding shows the complexity of interactions between genes which might affect swallowing neurophysiology in health and disease.

The most recent study in this literature used, for the first time, a more global approach and analysed over 500,000 SNPs from across the genome and their association with swallowing problems from individuals from the same well-characterised Dyne Steel Cohort [36••]. This analysis showed one SNP rs17601696 from an area of unknown function appeared to influence self-reported swallowing status. Genome-wide association studies are a useful tool in identifying possible genetic links and symptoms [10, 37]. One of the disadvantages of these studies is the need of replication of the results in multiple cohorts within different populations to provide more robust results.

Self-reported questionnaires, despite lowered accuracy, remain a useful tool for swallowing symptoms diagnosis. However, there are disadvantages of this tool such as: recall biases, silent aspiration, undetectable by individuals, and response biases (although response rates in this work were >80 %). Presented studies have predominantly used only one longitudinal cohort for analysis, and therefore replications in other cohorts are crucial. Studies conducted on humans are briefly summarised in Table 1.

**Table 1:** Summary of studies exploring genetics of swallowing physiology in humans

Gene	Technique	Cohort	Swallowing assessment	Author
*BDNF*	1 and 5 Hz transcranial magnetic stimulation	Healthy, young volunteers	MEPs with single pulse TMS	Jayasekeran et al. [14••]
	Oesophageal electrical stimulation	Healthy, young volunteers	Sensory (ST) and pain (PT) thresholds in the proximal (PE) and distal (DE) oesophagus	Vasant et al. [28]
	Pharyngeal electrical stimulation	dysphagic stroke patients	Validated dysphagia severity rating scale, recorded at baseline, 2 weeks and 3 months post recruitment	Essa et al. [29]
*APOE*	Association analysis	Elderly volunteers	Self-reported questionnaire	Mentz et al. [30]
*BDNF* + *COMT*	Association analysis	Elderly volunteers	Self-reported questionnaire	Nimmons et al. [50•]
rs17601696	Genome-Wide Association Study	Elderly volunteers	Self-reported questionnaire	Raginis-Zborowska et al. [36••]

## Genetics of swallowing—evidence from animal studies

Methodological issues around recruitment and detailed investigation and variability within the outcomes linked to human studies make animal studies an informative source of the genetic data associated with swallowing, in spite of their limitations (summarised in Table 2).

**Table 2:** Summary of studies exploring genetics of swallowing physiology in animals

Gene	Technique	Cohort	Swallowing assessment	Author
*UCHL1* *UCHL3*	Mice homozygous for both genes	Genetically modified mice	Presence of partially consumed, not digested food pellets in cages	Kurihara [38]
*PINK*	Knockout for *PINK1* rats	Adult Long-Evans rats	Tongue force, biting	Grant [41]
*BDNF* *TRKB*	Injection of *BDNF* into the dorsal vagal complex and repetitive electrical stimulation of the superior laryngeal nerve	Adult male Wistar rats	Measure of number of repetitive swallows	Bariohay [40]
	Tongue exercise effects on neurotrophic factors in the cranial sensorimotor system measured with immunoreactivity	Wistar rats	Tongue press task, immunoreactivity of *TRKB* in the sensorimotor system	Schaser [41]
*LEP (ob)*	Leptin microinjected at the subpostremal level of the medullary solitary tract nucleus	Adult Wistar rats	Swallowing rhythm recorded with electromyography	Felix [42]
*ERK*	Orofacial stimulation, immunohistochemical features in brainstem neurons, brainstem lesioning and of microinjection of GABA receptor agonist or antagonist into the nucleus tractus solitarii	Adult Sprague–Dawley rats	EMG activity was recorded	Tsujimura [43]

Kurihara et al. [38] examined the influence of two hydrolases encoded by genes *UCHL1* (OMIM 191342) and *UCHL3* (OMIM 603090) on dysphagia in mice. The authors reported that double-homozygous mice for both *UCHL1* and *UCHL3* had a 45 % weight reduction compared to the wild type which they used as a proxy for a direct measurement of dysphagia. They used the method of identification of undigested, but masticated food in the animals’ cages. The loss of weight could have different causes. A further limitation is that the authors examined only the pathological changes in the nucleus tractus solitarius (NTS), not examining the cerebral cortex. The presence of protein aggregation in the mouse’s brains might be evidence of neurological causes of swallowing impairments in these animals.

Other sources of potentially relevant genes have been examined in animal studies where weight has been assessed in Parkinson’s disease (PD) models. Over 80 % of PD patients develop swallowing impairments. Despite the developing diagnostic tools to measure swallowing impairment in rodents, such as videofluoroscopy, these techniques are not used in genetic studies of PD models in rats. A recent study explored the effects of mice knockout for *PINK1* gene (OMIM 608309) [39]. Limb and vocational function and neurodegenerative pathologies with immunochemistry were examined. Among other findings, the authors showed that, during the tongue protrusion task, knockout for *PINK1* gene rats had to use greater force in the ‘licking challenge’ and showed more variable licking patterns compared to wild-type rats. The main disadvantage was lack of more accurate swallowing impairment measures.

There are a number of studies exploring the effects of protein products of *BDNF, TRKB, OB, ERK* genes on swallowing physiology in rats [40–43].

The *BDNF* gene was examined in animal models, with the linkage to *TRKB* gene (also called *NTRK2*) [40, 41]. *TRKB* (OMIM 600456) gene encodes a member of the neurotrophic tyrosine receptor kinase (NTRK) family. Bariohay et al. [40] showed that *BDNF* inhibits the swallowing reflex in rats. Injection of *BDNF* into the dorsal vagal complex resulted in inhibition of regular swallowing induced by electrostimulation. Moreover, the inhibition is probably stimulated by the interaction of *BDNF* and GABAegric interneurons and is associated with *TRKB* activation. Bariohay’s studies overlook the impact of cortical areas while focusing only on the dorsal vagal complex (DVC) and its effect on swallowing. Other limitations include methodological problems in clearly showing that dysphagia in the rat is homologous to humans. The author’s conclusions were based on the presence of masticated, but not digested, food in the rats’ cages.

Comparatively, Schaser et al. [41] in a rodent model used 48 rats divided into three age groups. Immunocytochemistry tests showed that immunoreactivity of *TRKB* in the sensorimotor system decreases with age. Additionally, *BDNF* expression increased after tongue pressure exercises, but only in the young rats. Among the group of old and middle-aged rats, there were no significant decreases of immunochemistry of this protein. Moreover, there were no significant increases in *TRKB* and *BDNF* expression after tongue muscle exercises in old and middle-aged animals. These studies were only preliminary and further, more detailed investigation is needed.

Another protein which has been reported to possibly affect swallowing control is leptin, encoded by the *OB* gene. Leptin plays a role in the regulation of feeding behaviour. Felix et al. [42] showed the inhibitory effect of the *OB* gene (OMIM 164160) on swallowing in rats. The results showed effects of leptin on the swallowing central pattern generator (SwCPG) as well as motor neuron activity (motor outputs). Dysphagia in rats was diagnosed in the same way as in previous studies, that is, the presence/absence of masticated, undigested food. In terms of limitations, the authors were examining swallowing in general, not specifically dysphagia; the effects on appetite were excluded. Moreover, there has been no confirmation of these studies since 2006.

Swallowing difficulties were also studied in terms of orofacial pain that often occurs with dysphagia. Tsujimura et al. [43] investigated the effects of orofacial stimulation on the swallowing reflex, phosphorylated extracellular signal-regulated kinase (pERK) within the area of the nucleus tactus solitarius (NTS). Anaesthetised rats had stainless steel wire electrodes placed in the mylohyoid muscle to record EMG activity. Changes in swallowing performance were assessed by laryngeal movement and by mylohyoid electromyographic (EMG) activity. The findings provided evidence that facial pathways between the skin and NTS, as well as lingual muscles and the NTS, might modulate the swallowing reflex. While this study was not focused on the genetics of swallowing, it may provide some evidence for involvement of the gene encoding the pERK protein. As before, these studies examined only the involvement of the brainstem and not cortical areas in control of swallowing. A main advantage of the study was the more reliable and detailed method of swallowing assessment.

Animal studies from the field of neuroscience, despite some cohort replication advantages, carry different kinds of other disadvantages and, as such, interpretation of the results should be made with caution. One of the potential causes may be differences in brain structures even among the same species [34]. One of the advantages of using rats as the animal model is that they have a short life span (36 months), allowing study of ageing-related physiological changes, and responsiveness to different kinds of interventions.

Despite the limitations, a significant proportion of results from animal models can go on to replication in human studies; therefore, replication of the genetic loci from this work is warranted in future experimental work.

## Genetic syndromes, where one of the features is dysphagia

Dysphagia is a common symptom observed in congenital genetic syndromes. Studies conducted on patients with these genetic syndromes, where the detailed genetic background is examined, may provide another source of valuable information on swallowing genetics. The literature describing these complex genetic diseases could provide evidence about the chromosomal localisation of genes which may play a role in swallowing difficulties.

The following sections exclude syndromes where swallowing difficulties are caused by severe cleft palate (frequently observed in Pierre Robin Syndrome), inappropriate mastication and eating quickly which can cause choking (e.g. Prader–Willi syndrome), or single case studies.

## Potocki–Lupski syndrome—Ch 17 (dup(17)(p11.2p11.2)

Potocki–Lupski syndrome (PTLS) is caused by micro-duplication of chromosome 17p11.2 [dup(17)(p11.2p11.2)]. The phenotype is characterised by a number of dysmorphic features, hypotonia, sleeping problems, cardiovascular diseases and gaining insufficient weight. Moreover, patients suffer from neurological and cognitive features including intellectual impairment and autism. However, not every patient presents all of these features. Genetically, patients have duplication of a region of the short arm of chromosome 17.

Soler-Alfonso et al. [35] published studies about the association of oropharyngeal dysphagia and failure to thrive in PTLS. A limitation of this study was that swallowing function data were available from only 18 patients for study analysis. This is understandable, given that the disease is extremely rare. Another limitation was the method of dysphagia identification based on radiographic views of chewing and swallowing.

## Stuve–Wiedemann syndrome—locus 5p13.1

Stuve–Wiedemann syndrome (SWS) is a rare, genetic autosomal recessive disease characterised by bone dysplasias, respiratory distress, physical disability and early mortality. Most of the patients suffer from swallowing difficulties and resulting aspiration pneumonias which are a key contributor to death among these children [44].

Dagoneau et al. [45] investigated 19 families of SWS patients. Using a linkage analysis, the authors screened 24 patients with SWS from 19 families and revealed that chromosomal region 5p13.1 may be involved in the pathogenesis of this syndrome. Moreover, they analysed in more detail one of the genes from chromosome 5q13.1—*LIFR* and analysed the mRNA transcripts. Most of the children from analysed families had swallowing problems with diagnosed dysphagia. Another study on a two-year-old female with SWS and severe dysphagia confirmed the mutation in *LIFR* gene (OMIM 151443) [44]. However, *LIFR* is probably not directly associated with swallowing difficulties because its main function is in bone formation. Thus, further analysis of other genes from the 5p13.1 chromosomal region should be considered.

## CHARGE syndrome—locus 8q12

CHARGE syndrome is a mnemonic for coloboma of the eye, heart defects, atresia of the choanae, retarded growth and development, genital and/or urinary abnormalities, and ear anomalies. CHARGE syndrome is most likely caused by mutations within the chromosomal region 8q12. The main features of CHARGE syndrome comprise coloboma (abnormality of the eye caused by the missing tissue of the iris or the retina or the choroid), one- or two-sided choanal atresia (blocking of the nasal passage), cranial nerve dysfunction causing hearing and swallowing impairment, orofacial clefts, developmental delays and cardiovascular problems.

One study indicated that swallowing problems affect 79 % of children with CHARGE syndrome [46]. The swallowing impairment was assessed by parental reporting. A proportion of the cases with swallowing impairment may be caused by the clef palate which occurs in 20 % of children affected by the syndrome. Nevertheless, swallowing difficulties lead to more severe feeding difficulties which remain the leading cause of neonatal death in CHARGE syndrome. The main gene related to CHARGE syndrome is the *CHD7* gene (OMIM 608892) from the chromosome 8q12 which encodes Chromodomain Helicase DNA Binding Protein. The exact mechanisms of the pathways with *CHD7* gene remain unknown. Again, self-reported swallowing impairments should be evaluated with more accurate swallowing diagnostic tools with healthcare professionals.

## DiGeorge syndrome—locus 22q11

DiGeorge syndrome is caused by a small deletion of the chromosome 22q11. Clinical features are difficult to describe and vary between all individuals, even within the same family. The main features include heart defects and orofacial abnormalities. Patients with DiGeorge syndrome develop autoimmune disorders such as rheumatoid arthritis, breathing and hearing impairments, seizures caused by low levels of calcium, and gastrointestinal problems such as dysphagia [47].

VFS studies performed on 75 children with DiGeorge syndrome [48] identified problems with coordinating the suck/swallow/breath pattern leading to gagging or regurgitation. Karpinski et al. [49] recently developed an animal model of DiGeorge syndrome; 22q11 knockout mice were compared with mice with normal genotype and 21 genes (including *COMT* gene) were selected for the analysis. Apart from features such as altered jaw morphology, mice had swallowing impairments and chest infections caused by aspiration. Swallowing problems and aspiration were assessed following death by the presence of milk in the nose and the sinuses of infant mice. This may be a limitation of the study, because swallowing impairment assessment in mice and rats is problematic. Mice pups also had disrupted development of cranial nerves (CN) crucial for feeding and swallowing (CN X, CN IX, CN X). Different expression with knockout mice and wild type was observed in the *COMT* gene, and therefore may play a role in cortical plasticity in humans.

In the syndromes above, there are major genetic contributions to the clinical phenotypes. There are also well-specified regions of the genome implicated as causal in the problems that patients experience, including swallowing. One of the limitations of this research is the fact that swallowing problems within the cohorts of patients with congenital syndromes might be due to brainstem and/or other multifactorial/anatomical problems, with no evidence of cerebral cortex involvement. Nevertheless, the genetic loci implicated in this work should be considered in future experimental work.

## Conclusions

This review has explored a number of studies investigating genetic determinants of swallowing physiology and pathophysiology and possible responsiveness to treatment. Due to its complicated physiology, swallowing is most likely controlled by numerous genes and associated pathways. There are, of course, limitations to the available research. For example, studies using a candidate genetic analysis experimental approach are limited by the a priori choice of the genetic marker. As we have limited understanding in the mechanisms involved in neurogenic dysphagia, the choice of a genetic marker is often made by extrapolating information from associated phenotypes which may not be accurate. Nonetheless, the presented studies show the need for more comparative, integrative research protocols, consistency of methodological approaches and replication of existing findings in order to identify robust genetic candidates that may contribute to the neurophysiology of swallowing.
